# High-isolation antenna array using SIW and realized with a graphene layer for sub-terahertz wireless applications

**DOI:** 10.1038/s41598-021-87712-y

**Published:** 2021-05-13

**Authors:** Mohammad Alibakhshikenari, Bal S. Virdee, Shahram Salekzamankhani, Sonia Aïssa, Chan H. See, Navneet Soin, Sam J. Fishlock, Ayman A. Althuwayb, Raed Abd-Alhameed, Isabelle Huynen, James A. McLaughlin, Francisco Falcone, Ernesto Limiti

**Affiliations:** 1 Electronic Engineering Department, University of Rome “Tor Vergata”, Via del Politecnico 1, 00133 Rome, Italy; 2 Center for Communications Technology & Mathematics, School of Computing and Digital Media, London Metropolitan University, London, N7 8DB UK; 3 Institut National de La Recherche Scientifique (INRS), University of Quebec, Montréal, QC H5A 1K6 Canada; 4 School of Engineering and the Built Environment, Edinburgh Napier University, Edinburgh, UK; 5 School of Engineering, Ulster University, Newtownabbey, Belfast, Northern Ireland BT37 0QB UK; 6 Electrical Engineering Department, Jouf University, Sakaka, 72388 Saudi Arabia; 7 Faculty of Engineering and Informatics, University of Bradford, Bradford, UK; 8 Institute of Information and Communication Technologies, Electronics and Applied Mathematics, Université Catholique de Louvain, Ottignies-Louvain-la-Neuve, Belgium; 9 Electric, Electronic and Communication Engineering Department, Public University of Navarre, 31006 Pamplona, Spain; 10 Institute of Smart Cities, Public University of Navarre, 31006 Pamplona, Spain

**Keywords:** Engineering, Electrical and electronic engineering

## Abstract

This paper presents the results of a study on developing an effective technique to increase the performance characteristics of antenna arrays for sub-THz integrated circuit applications. This is essential to compensate the limited power available from sub-THz sources. Although conventional array structures can provide a solution to enhance the radiation-gain performance however in the case of small-sized array structures the radiation properties can be adversely affected by mutual coupling that exists between the radiating elements. It is demonstrated here the effectiveness of using SIW technology to suppress surface wave propagations and near field mutual coupling effects. Prototype of 2 × 3 antenna arrays were designed and constructed on a polyimide dielectric substrate with thickness of 125 μm for operation across 0.19–0.20 THz. The dimensions of the array were 20 × 13.5 × 0.125 mm^3^. Metallization of the antenna was coated with 500 nm layer of Graphene. With the proposed technique the isolation between the radiating elements was improved on average by 22.5 dB compared to a reference array antenna with no SIW isolation. The performance of the array was enhanced by transforming the patch to exhibit metamaterial characteristics. This was achieved by embedding the patch antennas in the array with sub-wavelength slots. Compared to the reference array the metamaterial inspired structure exhibits improvement in isolation, radiation gain and efficiency on average by 28 dB, 6.3 dBi, and 34%, respectively. These results show the viability of proposed approach in developing antenna arrays for application in sub-THz integrated circuits.

## Introduction

Wireless communication bands are increasingly becoming highly crowded and the ever-increasing demand for more bandwidth has provoked the exploitation of the yet unexplored electromagnetic spectrum in the sub-terahertz (THz) region^1^. Higher bandwidth afforded by the sub-terahertz band can achieve extremely high data-rates in the order of 0.1 THz/s for future wireless systems^2^. However, sub-terahertz radiation is absorbed by most materials, including the molecules in the atmosphere such as water vapor. For such systems, line-of-sight propagation is needed and even for such conditions, the range is rather limited due to the inherently high path-loss. Since path-loss is relatively high and the current sub-THz receivers are not sensitive, hence much effort needs to be devoted on maximizing both the radiation-gain and efficiency of sub-terahertz antennas.

Owing to the lack of sub-terahertz sources and detectors, this area of the electromagnetic spectrum has not been fully explored for wireless communication applications^3^. The up and down-converters as well as low-gain amplifiers constituting the RF front-end of sub-terahertz communication system have restricted output power and bandwidth. Existing technology relies on mixing optical sources essentially lasers to generate low to moderate power-levels at sub-THz band. Receivers at such frequencies rely on direct detection using Schottky-diodes, which is not band selective and has a low dynamic range and sensitivity^4^.

At sub-THz frequencies metals exhibit a lower conductivity than at DC. This phenomenon enhances field infiltration within the metal at sub-THz and adversely affect the radiation efficiency of metallic antennas^5^. In addition to the low conductivity of metal at these frequencies, the effect of small geometric parameters of metallic antennas, specifically the width or radius of metallic traces which are smaller than 0.1 micron, must be considered. This is because numerical analysis reveals that small scale antennas of sub-100 nano-meters compared to their millimeter-sized counterparts results in significantly low radiation efficiency at sub-THz frequency due to the high surface resistance of metallic traces^6^. Moreover, because of the method employed for conductor deposition in the fabrication of ultra-thin metallic traces of sub-nano-meter thickness, the magnitude of the metal’s conductivity becomes considerably lower than that of the bulk material. The low conductivity is attributed to the following factors: grain boundary scattering, surface scattering, and surface roughness^7, 8^. These factors can considerably reduce the theoretically predicted radiation efficiency of antennas at sub-terahertz frequencies.

Recently various sub-THz antenna arrays have been investigated for THz spectroscopy based on slots^9^, printed dipoles^10^ or patch antennas^11^ however the radiation characteristics of these antennas are limited for practical applications. Hence there is demand for antennas at these frequencies with improved radiation gain and efficiency characteristics. Although high performance wideband horn antennas are now available^12^, they are bulky in structure even at very high frequencies. Instead, low profile planar antennas are desirable for ease of integration with RF-circuits to realize compact transceivers^13, 14^.

In this study the isolation, radiation-gain and efficiency of sub-terahertz array structure is improved by employing a combination of substrate integrated waveguide (SIW) and metamaterial (MTM) inspired technologies. The prototype antenna arrays were designed and analysed using numerical simulation tools, and the optimized designs fabricated and measured. The array structures were coated with a thin layer of Graphene as it supports the propagation of surface plasmon polaritons enabling it to operate in the sub-terahertz frequency band^15, 16^. In^17^ it has been shown that plasmons in graphene layer strongly confine electromagnetic energy, and in^18^ it has been shown that the application of a voltage to the graphene layer affects the phase characteristics of the antenna. These results show Graphene material opens exciting prospects for antenna development in the terahertz frequency band.

## Sub-terahertz antenna based on SIW Technology

**Figure 1:**
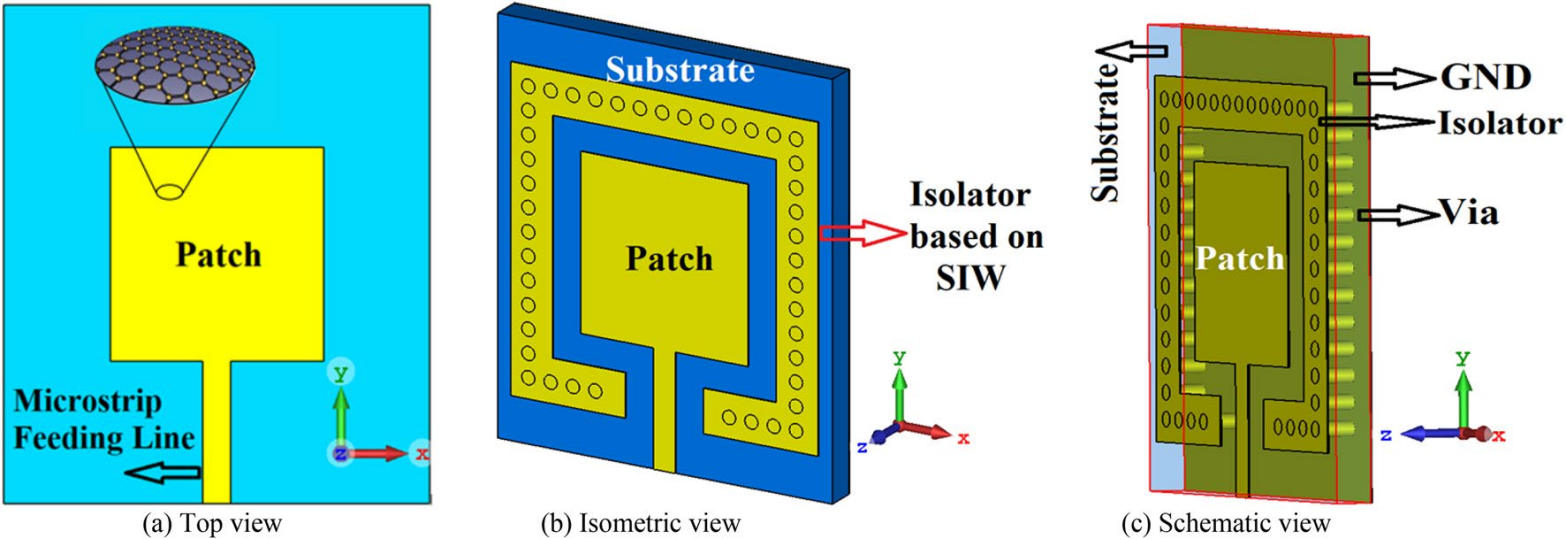
Antenna structure: (**a**) reference antenna with no SIW technology, (**b**) and (**c**) the proposed antenna with SIW technology.

**Figure 2:**
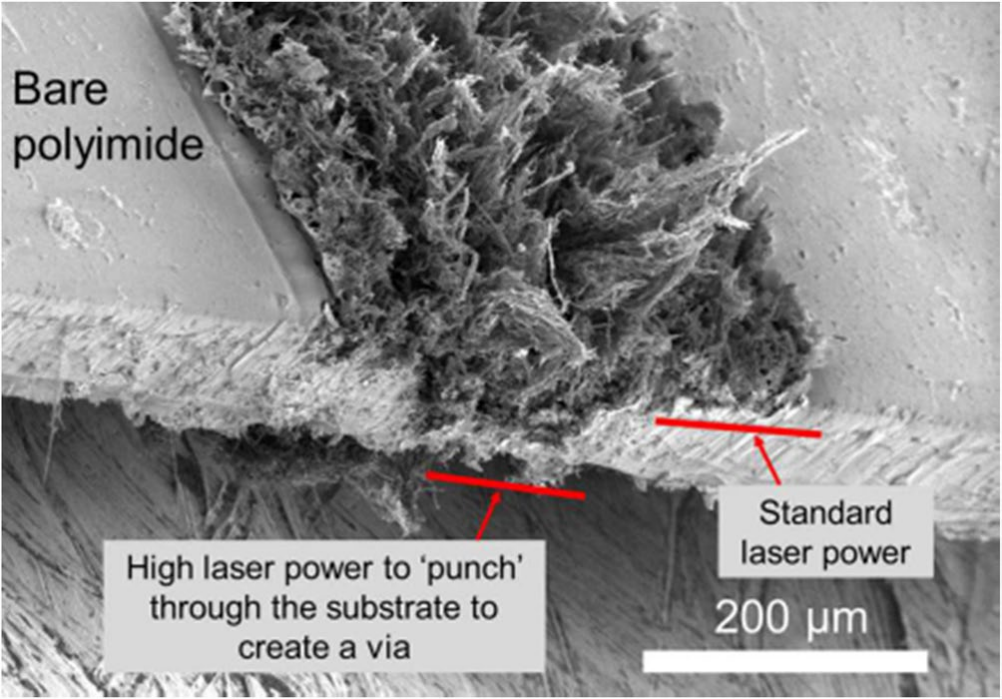
Scanning electron microscope (SEM) image of polyimide substrate (65º tilt angle) to highlight how the vias are fabricated by increasing the scribing laser power to ‘punch’ through the substrate and create an electrically conducting through-connection.

**Figure 3:**
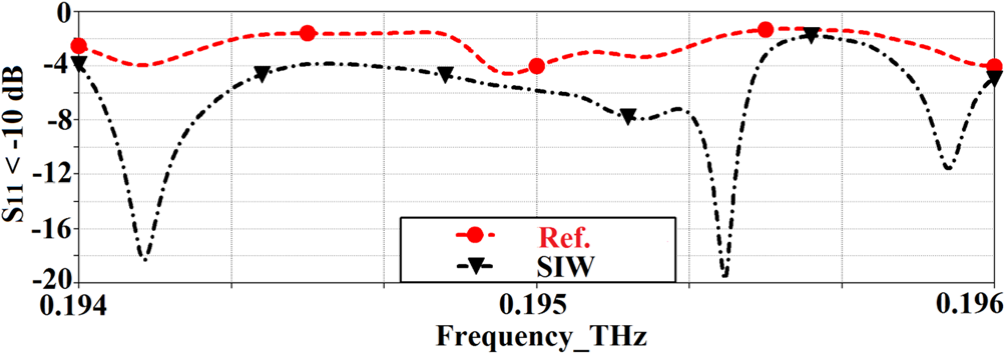
Simulated reflection-coefficient response of the reference and SIW-loaded patch antennas.

**Figure 4:**
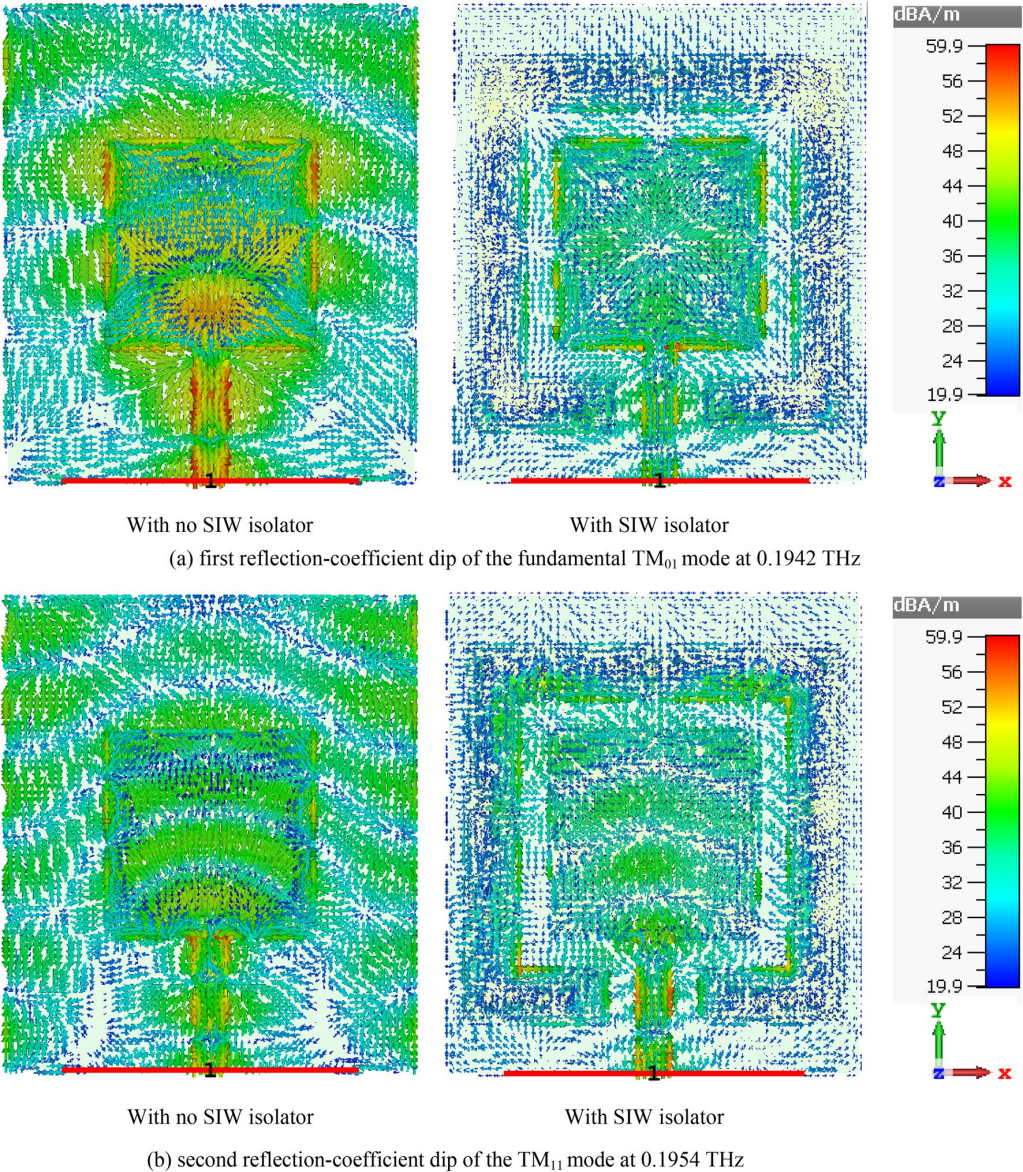
Surface current density distributions over the reference and SIW antennas at the first and second reflection-coefficient dip frequencies in Fig. 2.

**Figure 5:**
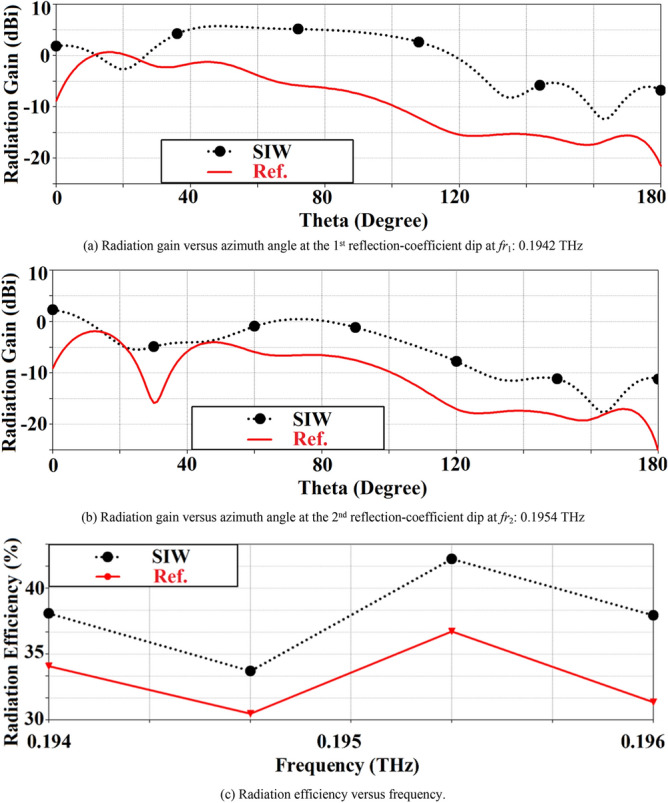
Simulated radiation gain and efficiency plots of the reference and SIW-loaded antennas.

Geometries of the standard reference patch and the proposed SIW-loaded patch antenna are shown in Fig. 1. The patch antenna was constructed on a polyimide dielectric substrate (Kaneka, Apical NP, USA) with thickness of 125 μm, dielectric constant (${\varepsilon }_{r}$) of 3.5, and loss-tangent (tang$\delta$) of 0.0027. To produce the graphene patch, a CO2 laser (Universal Laser 230 VLS) was used to scribe the front and rear polyimide surfaces and thereby convert this into a graphene film, using the same experimental conditions as discussed in^19^. To produce the vias, the laser power was increased from 8.1 W (as used for the standard patch on the front face) to 15 W, and the laser scribe was repeated twice in these areas, in order to ‘punch’ through the polyimide surface. A cross section of the via taken using an SEM (Hitachi SU5000 field emission scanning electron microscope) can be observed in Fig. 2. The proposed antenna includes SIW isolator which is framed around the square patch. Dimensions of the antenna are given in Table 1. Reflection-coefficient response for both antennas over 0.194–0.196 THz is shown in Fig. 3. The results reveal SIW isolation improves the impedance match of the antenna, which is pronounced at the resonance frequencies of 0.1942 THz and 0.1954 THz. Surface current density distribution over the two antennas at their dominant resonant modes in Fig. 4 show the effectiveness of the SIW isolator in suppressing the propagating surface waves over the structure. Suppression of surface waves is desirable in antenna arrays to mitigate mutual coupling between adjacent radiating elements and thereby improve the antenna’s radiation characteristics. Radiation gain and efficiency plots of both antennas at the resonant frequencies in Fig. 5 show the SIW isolator significantly enhances the radiation-gain and efficiency properties over the reference antenna, due to reduction in substrate loss.

**Table 1:** Dimensions of the antenna.

Parameter	Dimension (mm)
Patch length	3
Patch width	3
Feedline length	2
Feedline width	0.4
SIW isolator width	0.75
Gap between patch and isolator	0.5
Gap between feedline and isolator	0.5
Diameter of vias	0.25
Gap between vias	0.20
Thickness of graphene layer	0.0005
Substrate thickness	0.125

## SIW loaded antenna with slots

**Figure 6:**
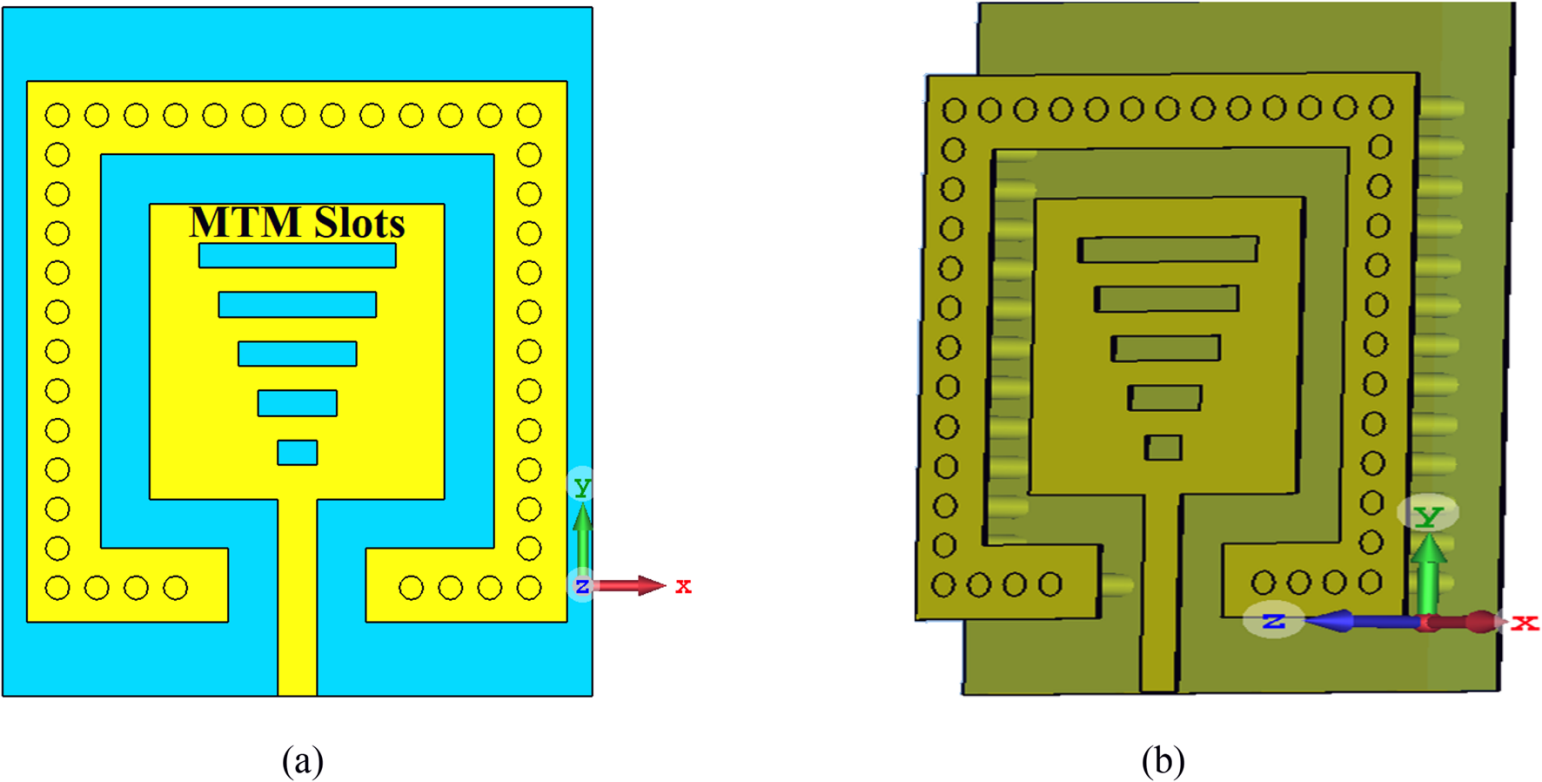
(**a**) front view, (**b**) schematic view of the SIW-loaded slotted antenna.

**Figure 7:**
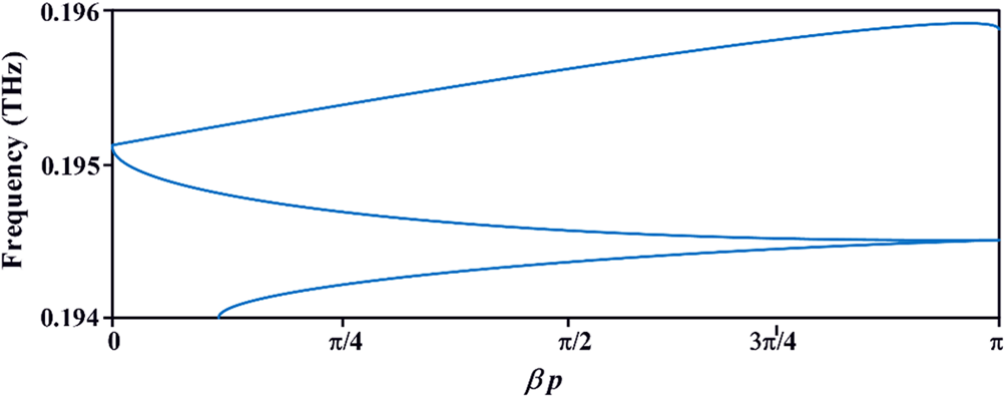
Dispersion diagram of the SIW-loaded slotted antenna.

**Figure 8:**
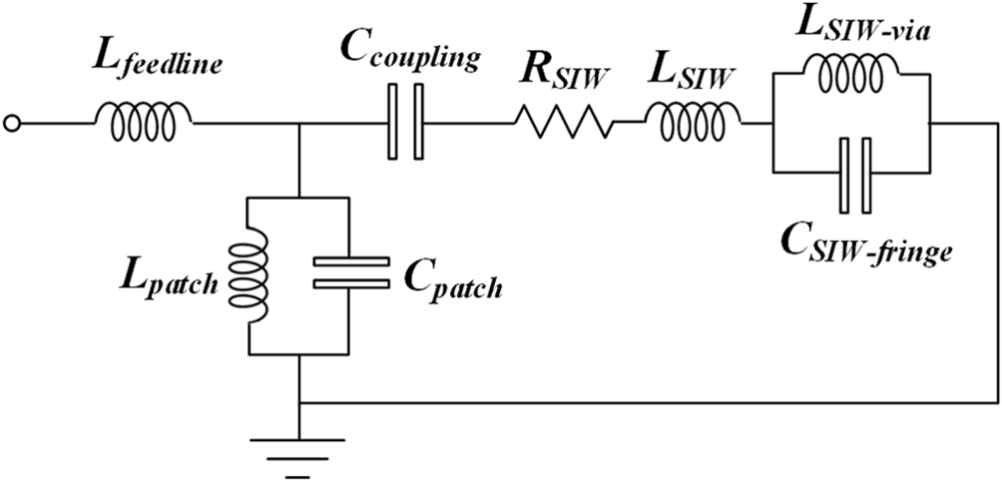
The simplified equivalent circuit of the SIW-loaded slotted antenna.

Metasurface characteristics are introduced into the patch antenna by the inclusion of periodic array of sub-wavelength length slots of varying length to realize a wider impedance bandwidth, as shown in Fig. 6^20^. These slots exposing the substrate essentially manipulate the electromagnetic (EM) response of the surface. The EM waves impinging on the metasurface induce electric and magnetic dipole moments, which are related to the effective permittivity and permeability of the composite medium. Detailed theoretical analysis of this type of structure is provided in^21^. To confirm the antenna supports backward waves the CST Microwave Studio, which is a 3D electromagnetic simulation tool, was used to obtain its dispersion characteristics that was computed using^22^:

1 $$\beta p = cos^{ - 1} \left( {\frac{{1 - S_{11} S_{22} + S_{12} S_{21} }}{{2S_{21} }}} \right)$$

where *β* is the wave propagation constant along the direction of propagation, and *p* is the length of the unit-cell. Figure 7 shows how the phase through the structure varies with frequency. The dispersion diagram slope is negative between 0.1945 and 0.1951 THz. Over this frequency range the signal group and phase velocities are oriented along opposite directions resulting in a backward wave, which is characteristic of metamaterials. This is due to the sub-wavelength slots that control the electromagnetic response of the surface to produce a homogeneous response at 'macroscopic' level. The simplified equivalent circuit of the SIW-loaded slotted antenna in Fig. 8 will be referred to hereon as SIW-loaded MTM. The lumped element values in the equivalent circuit were extracted using the well-established pseudo-inverse technique from S-parameters^22, 23^. According to^22^ this structure essentially acts like MTM or composite right/left-handed (CRLH) structure. Magnitude of the equivalent circuit parameters are: *L*_*feedline*_ = 0.021 pH, *L*_*patch*_ = 1.74186 pH, *C*_*patch*_ = 0.408236 pF, *R*_*SIW*_ = 35 Ω, *L*_*SIW*_ = 1 pH, *L*_*SIW-*via_ = 2.44918 pH, & *C*_*SIW-fringe*_ = 0.204575 pF.

The array of periodic slots etched on the patch generate multiple resonances whose frequencies can be determined from the dispersion curve when the following condition is satisfied^20^:

2 $$\beta p = \frac{n\pi }{M}\left\{ {\begin{array}{*{20}l} {n = 0, \; \pm 1, \ldots , \; \pm \left( {M - 1} \right),} \hfill \\ { {\text{for}}\;{\text{T - type unit - cell}}} \hfill \\ {n = 0, \; \pm 1, \ldots , \; \pm \left( M \right), } \hfill \\ {{\text{for }}\;\uppi {\text{ - type}}\;{\text{unit - cell}}} \hfill \\ \end{array} } \right.$$

**Figure 9:**
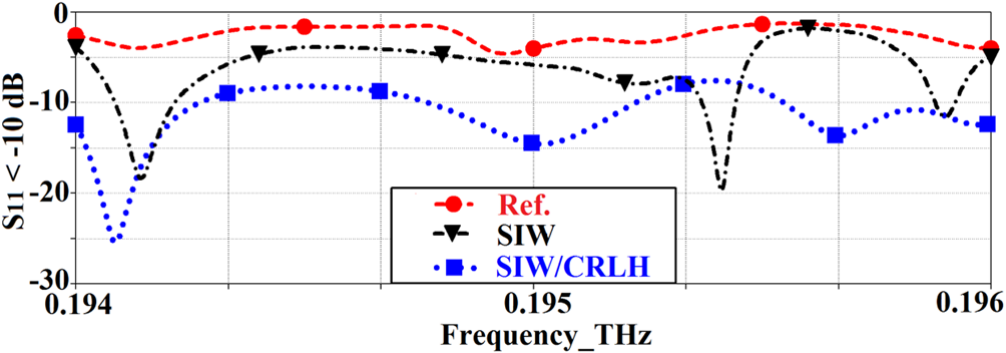
Simulated reflection-coefficient response of the reference, SIW-loaded, and SIW-loaded MTM (CRLH) antennas.

It can be shown from Eq. (2), there are three resonance frequencies which can be excited including the ± 1st-order resonances and the zeroth-order resonance at *f*_*se*_. These resonant frequencies are verified from the reflection-coefficient response of the antenna in Fig. 9, which are at *f*_*r−*1_: 0.1941 THz, *f*_*r0*_: 0.1950 THz, and *f*_*r*+*1*_: 0.1957 THz. The reflection-coefficient shows significant improvement in impedance match between 0.194 and 0.196 THz with the inclusion of the slots.

**Figure 10:**
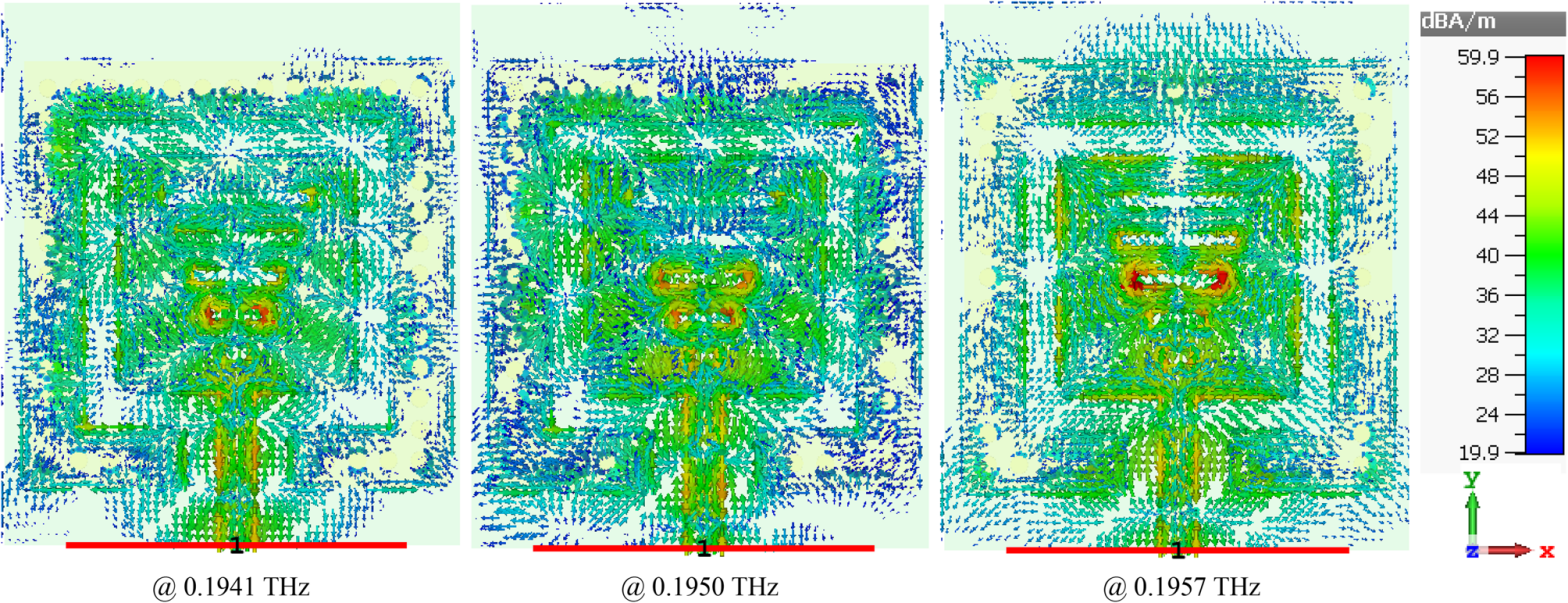
Surface current density distributions over the SIW-loaded MTM antenna at the first, second and third resonance frequencies.

The current density distribution in *xy*-plane at the three resonant frequencies is shown in Fig. 10. The current distribution at *f*_*r−*1_: 0.1941 THz and *f*_*r*+1_: 0.1957 THz are very similar; however, it is significantly muted at *f*_*r0*_: 0.1950 THz. The size of the antenna is 3.89λ_0_ × 4.54λ_0_ × 0.08λ_0_ when operated at *f*_*r−*1_: 0.1941 THz.

**Figure 11:**
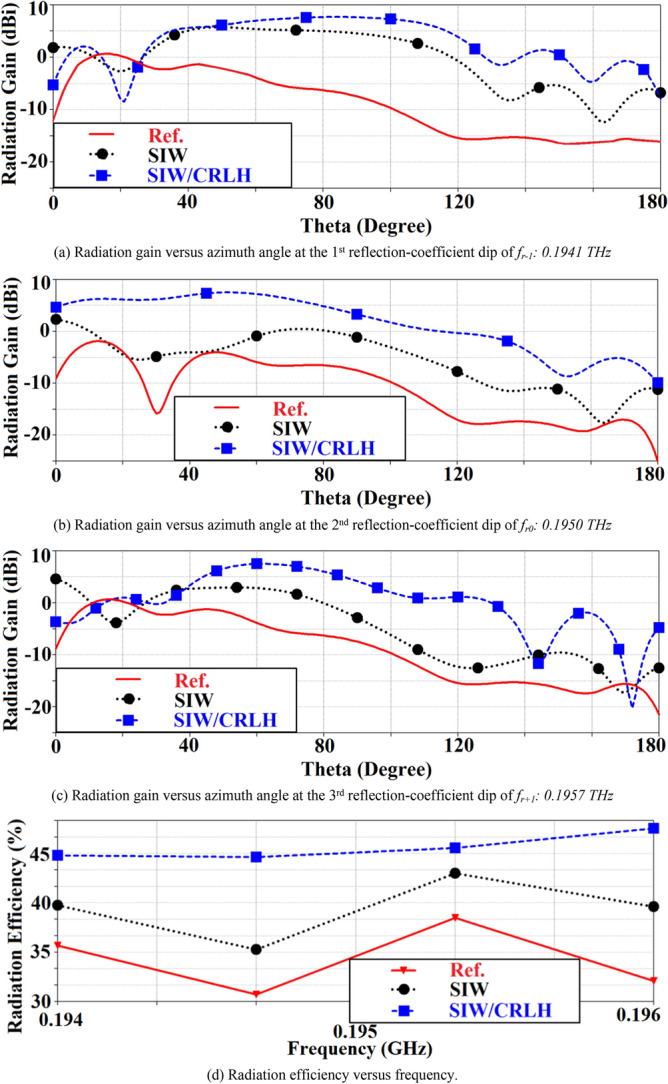
Simulated radiation gain and efficiency of the reference, SIW-loaded, and SIW-loaded MTM (CRLH) antennas.

Comparison of the radiation plots of the reference, SIW-loaded, and SIW-loaded slotted antennas are shown in Fig. 11. It is evident that the SIW-loaded MTM patch antenna exhibits significantly improved performance over the other two antennas. The optimum radiation gain of the SIW-loaded MTM antenna at resonance modes − 1st (*f*_*r-1*_: 0.1941 THz), zeroth (*f*_*r0*_: 0.1950 THz), and + 1st (*f*_*r*+1_: 0.1957 THz) are 8.05 dBi @87.7°, 8.0 dBi @51.6°, and 7.92 dBi @59.3°, respectively. Besides the gain, the radiation efficiency at resonance modes − 1st, zeroth, and + 1st are 45%, 45%, and 47%, respectively. In the case of *f*_*r−*1_: 0.1941 THz and *f*_*r*+1_: 0.1957 THz, the gain of the reference antenna is comparable to the other two antennas, but this is over a very narrow azimuth range between 12° and 26°. Total dimensions of the three antennas are identical.

## Array configuration of the proposed SIW-loaded MTM antenna for sub-THz applications

In this section 2 × 3 antenna arrays are realized based on the standard reference, SIW-loaded, and SIW-loaded MTM patch antenna. The main issue encountered in an array structure is the adverse effect of mutual coupling between the radiating elements constituting the array. Several techniques previously studied and proposed are exemplified in^23–28^ but the size of the array matrix investigated in all cases reported was limited to 1 × 2. The techniques proposed previously include the use of air gap between radiator and ground-plane or the implementation of ground-plane defection. In all cases reported the isolation achieved is limited and is restricted over narrow frequency band.

**Figure 12:**
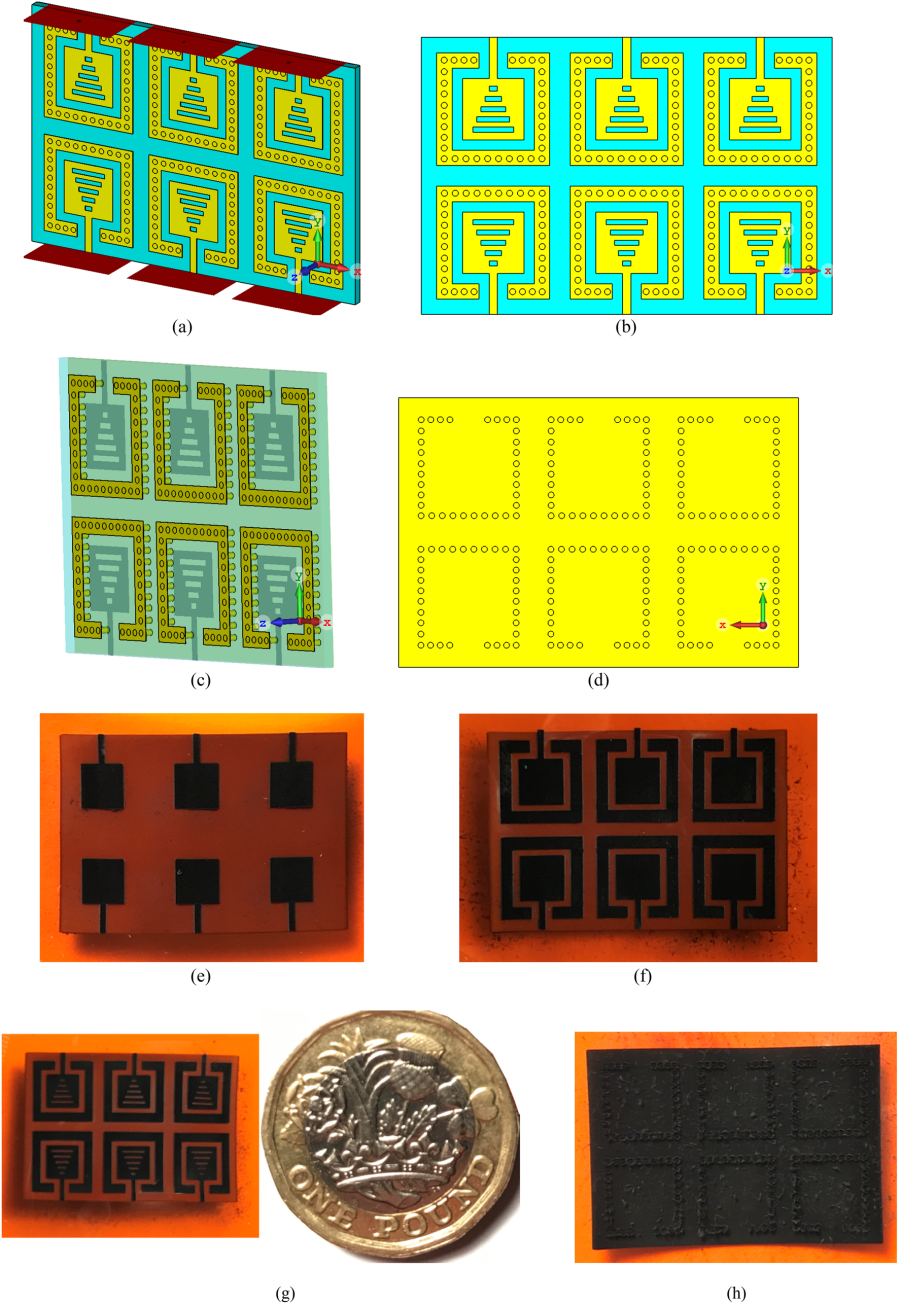
Proposed Graphene coated 2 × 3 antenna array based on SIW-loaded MTM patches, (**a**) isometric view, (**b**) front view, (**c**) SIW isolators framing the radiation patches, (**d**) back view (ground-plane), and (**e**–**h**) the fabricated antenna array prototype of the reference, SIW-loaded, SIW-loaded MTM antenna, and the ground-plane, respectively.

In small-sized antenna array structures it is very difficult to supress the mutual coupling effects resulting from surface wave interaction between the radiation elements due to their proximity. Proposed here is a simple but effective mutual coupling reduction technique based on SIW isolator as described in “SIW loaded antenna with slots” section. The prototype of the SIW-loaded MTM antenna is shown in Fig. 12, where the SIW isolator is wrapped around each patch to significantly reduce propagation of surface waves and minimise substrate loss. The prototype antenna array was coated with 500 nm thick film of Graphene. The identical patches in the array have dimensions of 3 × 3 × 0.125 mm^3^. The dimension of the 2 × 3 antenna array is 20 × 13.5 × 0.125 mm^3^. Dimensions of the optimized structural parameters are tabulated in Table 2.

**Table 2:** Structural dimensions of the antenna.

Parameters	Dimension (mm)
Patch length	3
Patch width	3
Feed line length	2
Feed line width	0.4
SIW isolator width	0.75
Number of tapered rectangular slots	5
Slot lengths (sub-wavelength)	0.48, 0.56, 0.84, 1.12, 1.40
Slot width	0.25
Gap between patch and isolator	0.5
Gap between feed line and isolator	0.5
Diameter of vias	0.25
Gap between vias	0.20
Total length	20
Total width	13.5
Thickness of graphene layer	0.0005
Substrate thickness	0.125
Edge-to-edge gap between adjacent patches	3.5
Edge-to-edge gap between SIW isolators	1

## Results and discussions

**Figure 13:**
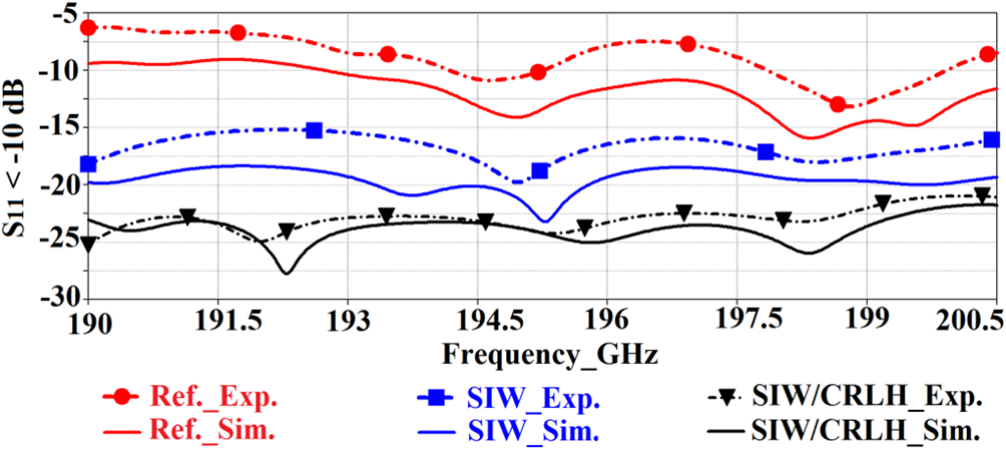
Simulated and experimental results of the reflection-coefficient of the reference, SIW-loaded, and SIW-loaded MTM (CRLH) antenna arrays.

The simulated and measured impedance bandwidth of the standard reference, SIW-loaded, and SIW-loaded MTM 2 × 3 antenna arrays are shown in Fig. 13. CST Microwave Studio was used obtain the simulation results. Keysight PNA Network Analyzer with a frequency extender was used to measure the reflection-coefficient of the antenna. These results clearly show that the SIW isolator has a significant impact on the impedance matching and bandwidth of the array over the standard reference antenna array. Further improvement is achieved by incorporating sub-wavelength slots in the patch antennas. The experimental results show that with SIW-loading the impedance matching is improved by 10 dB on average from 190 to 200 GHz over the reference antenna array. By incorporating both SIW-loading and sub-wavelength slots the impedance matching also improves on average by 14 dB from 190 to 200 GHz compared to the reference array antenna. There is generally good agreement between the simulated and measured results. The discrepancy in the results is attributed to manufacturing tolerance and unaccounted loss in the simulation model.

**Figure 14:**
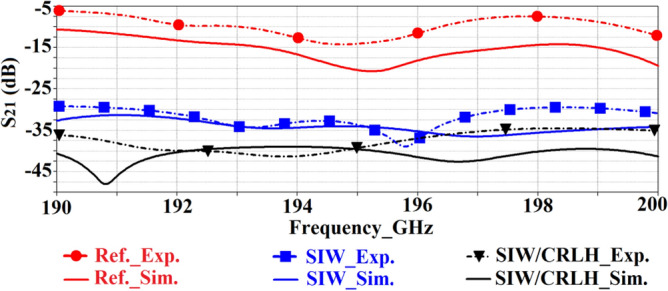
Simulated and experimental results of the transmission-coefficient (S_21_) of the reference, SIW-loaded, and SIW-loaded MTM (CRLH) antenna arrays.

The transmission-coefficient response of the standard reference, SIW-loaded, and SIW-loaded MTM 2 × 3 antenna array are shown in Fig. 14. These results demonstrate the effectiveness of the proposed technique of using SIW isolator in suppressing the surface-wave propagations and reducing substrate loss to mitigate mutual coupling effects without increasing the overall size of the array structure. Experimental results show that compared to the reference antenna array the SIW-loaded array is shown to improve the isolation on average by 17 dB from 190 to 200 GHz. Also, by incorporating sub-wavelength slots the SIW-loaded MTM array is shown to suppress mutual coupling on average by more than 28 dB across 190 GHz to 200 GHz compared to the reference array antenna.

**Figure 15:**
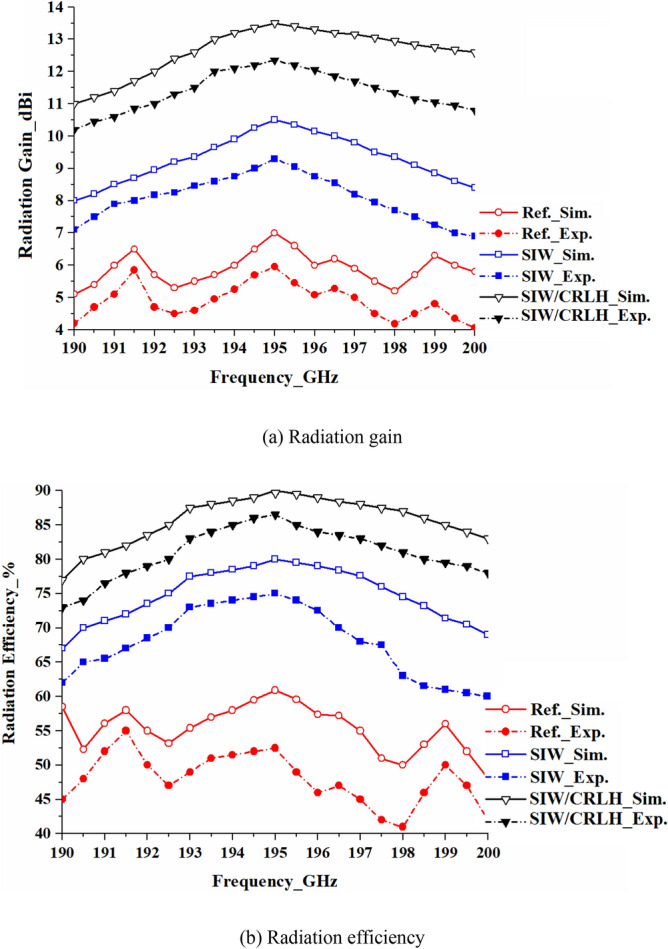
Simulated and experimental results of the radiation gain and efficiency as a function of frequency of the reference, SIW-loaded, and SIW-loaded MTM (CRLH) antenna arrays.

The simulated and measured radiation gain and efficiency of the standard reference, SIW-loaded, and SIW-loaded MTM (CRLH) array are shown in Fig. 15. Experimental results show that compared to the reference array the SIW-loaded array’s gain and efficiency improve on average by 2.8 dBi and 23%, respectively, between 190 and 200 GHz. With SIW-loaded MTM the average improvement in gain and efficiency over the reference array is 6.3 dBi and 34%, respectively, between 190 and 200 GHz. These results demonstrate the array’s effective aperture is enlarged by incorporating sub-wavelength slots, which has the benefit of not affecting the antenna’s dimensions.

**Figure 16:**
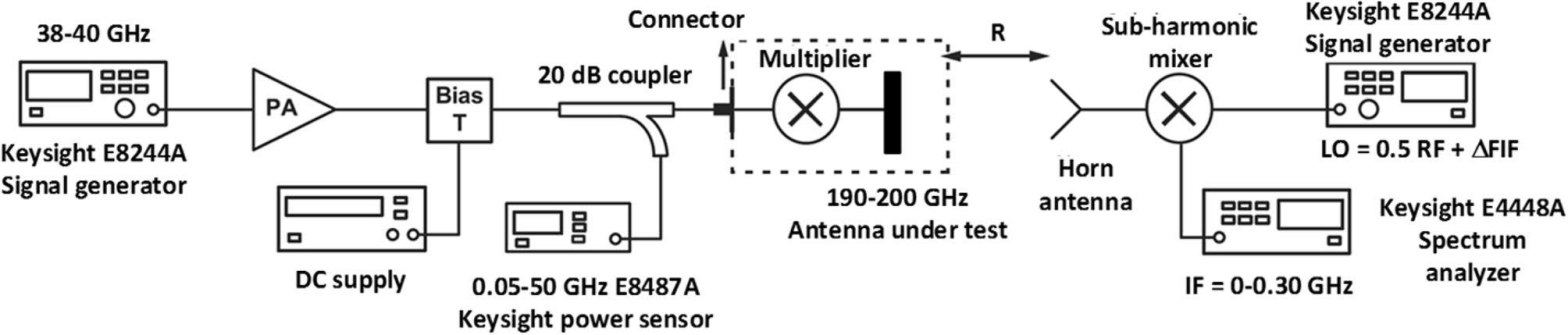
Set-up to measure the array’s radiation patterns.

**Figure 17:**
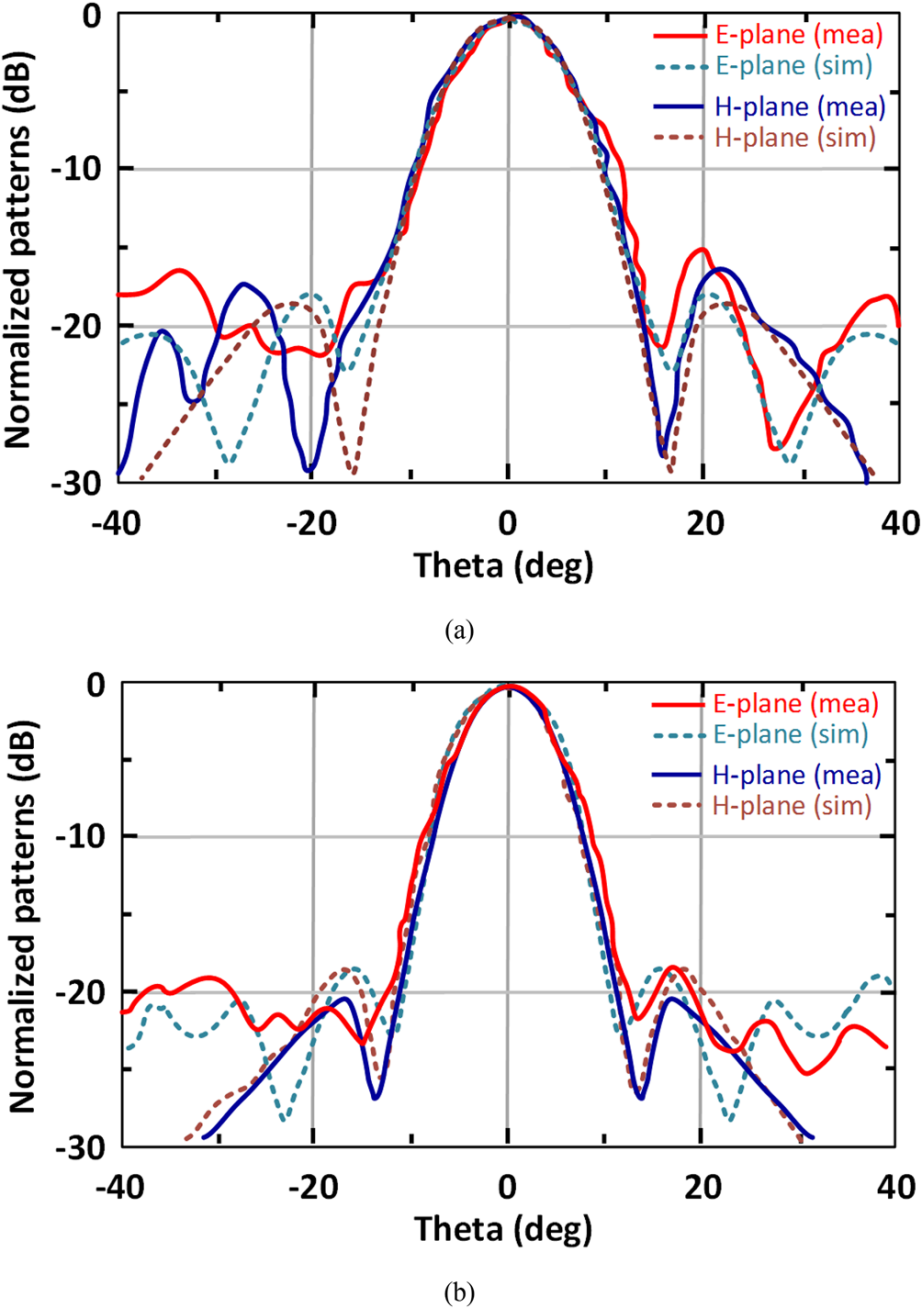
Measured and simulated radiation pattern of the 2 × 3 SIW-loaded MTM antenna arrays in E-plane and H-plane at (**a**) 190 GHz, and (**b**) 200 GHz.

The set-up to measure the far-field radiation patterns of the SIW-loaded MTM antenna arrays is shown in Fig. 16. In transmit mode we used a 2-way power splitter in conjunction with two 3-way power splitters. It was necessary to ensure the lengths were such that the phase at the antenna terminals were identical. This arrangement was used to split equally the input sub-THz signal applied to the 2 × 3 array. Conversely, in the receive mode we used two 3-way power combiners in combination with a 2-way power combiner. This arrangement combined the received signal power from the 2 × 3 array. The output from the array in receive mode was connected to Keysight E4448A spectrum analyser. Keysight E8244A signal generator was used to produce a signal that was adjustable in the frequency range 38–40 GHz with enough power (10–20 dBm) for the mixer to generate a set of harmonics simultaneously. The 5th harmonic was used as it was in the desired frequency range of 190–200 GHz. At the receiver, the received signal is mixed with signal from Keysight E8244A with a frequency which was offset from the transmitter local oscillator, and this offset frequency (ΔF_IF_) determines the system harmonic number, which is used (5ΔF_IF_). The simulated and measured patterns of the 2 × 3 SIW-loaded MTM antenna arrays in the E- and H-planes at 190 GHz and 200 GHz are shown in Fig. 17. CST Microwave Studio was used obtain the simulation results. The measured results agree well with the simulations. The radiation pattern is stable and symmetrical within the operating band of the array. Due to the unpredictable reflection from the fixtures and measurement setup near the antenna under test, a series of small ripples can be seen in the measured results.

Performance parameters of the proposed SIW-loaded MTM antenna array is compared with other recently published millimeter-wave and sub-THz antennas in Table 3. The comparison shows that the proposed antenna array has comparable gain and radiation efficiency to the cited references, which confirm the effectiveness of the proposed approach. It is less complex and cost effective to implement and realize in practice for mass production, which makes it a viable candidate for applications to sub-terahertz wireless systems.

**Table 3:** Salient features of the proposed SIW-loaded MTM antenna array compared with recent publications.

References	Type	Bandwidth [freq. range]	Gain (dBi)	Eff. (%)	Size (mm^3^)
^29^	Bowtie-slot	15 GHz [90–105]	Max − 1.78	–	0.71 × 0.31 × 0.65
^30^	Differential-fed	20 GHz [50–70]	Max − 3.2	–	1.5 × 1.5 × 0.3
^31^	Ring-shaped monopole	20 GHz [50–70]	Max. 0.02	Max. 35	–
^32^	Circular open-loop	10 GHz [57–67]	Max − 4.4	–	1.8 × 1.8 × 0.3
^33^	AMC embedded squared slot antenna	51 GHz [15–66]	Max. 2	–	1.44 × 1.1
^34^	Monopole	25 GHz [45–70]	Max. 4.96	–	1.953 × 1.93 × 0.25
^35^	Loop antenna	4 GHz [65–69]	Max. 8	Max. 96.7	0.7 × 1.25
^36^	Dipole-antenna	7 GHz [95–102]	Max. 4.8	–	–
^37^	Tab monopole	30 GHz [45–75]	Max. 0.1	Max. 42	1.5 × 1
^38^	Patch fed higher order mode DRA	25 GHz [330–355]	Max. 7.9	Max. 74	0.2 × 0.5
^39^	On-chip 3D (Yagi like concept)	40 GHz [320–360]	Max. 10	Max. 80	0.7 × 0.7 × 0.43
^40^	Half-mode cavity fed DRA	15 GHz [125–140]	Max. 7.5	Max. 46	0.8 × 0.9 × 1.3
^41^	Slot fed stacked DRA	10 GHz [125–135]	Max. 4.7	Max. 43	0.9 × 0.8 × 1.5
^42^	DRA	20 GHz [120–140]	Max. 2.7	Max. 43	0.9 × 0.8 × 0.6
^43^	8 × 8 Magneto-electric dipole antenna array	14.7 GHz [130.3–145]	Max. 20.5	Max. 59.2	32 × 20 × 0.818
^44^	4 × 1 Patch antenna array	32 GHz [259–291]	Max. 5.2	–	2.47 × 1.53 × 0.675
^45^	2 × 1 Octagonal shorted annular ring antenna array	17 GHz [303–320]	Max. 4.1	Max. 38	0.55 × 0.5 × 0.3
This work	SIW-loaded MTM	10 GHz [190–200]	Max. 12.2	Max. 86	20 × 13.5 × 0.125

## Conclusion

We have demonstrated the use of a Graphene-based antenna at sub-THz and the effectiveness of the proposed technique of framing each radiator with SIW. This technique is shown here to reduce surface wave propagations and suppressing near-field mutual coupling between the radiators in an array. The consequence of this is significant improvement in the array’s performance in terms of impedance matching and radiation performance which is achieved without compromising the footprint of the antenna array. Further improvement in performance was demonstrated by incorporating sub-wavelength slots in the patch radiators. The feed mechanism employed is however considered to be unfeasible for a large array, where aperture coupling would be more appropriate.
